# A Novel Cu(II)-Binding Peptide Identified by Phage Display Inhibits Cu^2+^-Mediated Aβ Aggregation

**DOI:** 10.3390/ijms22136842

**Published:** 2021-06-25

**Authors:** Xiaoyu Zhang, Xiancheng Zhang, Manli Zhong, Pu Zhao, Chuang Guo, You Li, He Xu, Tao Wang, Huiling Gao

**Affiliations:** 1 College of Life and Health Sciences, Northeastern University, Shenyang 110819, China; zhangxiaoyu@dicp.ac.cn (X.Z.); zhangxiancheng@dicp.ac.cn (X.Z.); zhongmanli@mail.neu.edu.cn (M.Z.); zhaopu6687700@mail.neu.edu.cn (P.Z.); guoc@mail.neu.edu.cn (C.G.); nanjiliyou@163.com (Y.L.); wt7294@hotmail.com (T.W.); 2 Key Laboratory of Separation Science for Analytical Chemistry, Dalian Institute of Chemical Physics, Chinese Academy of Sciences, Dalian 116023, China; 3 Department of Histology and Embryology, School of Medicine, Shenzhen University, Shenzhen 518060, China; oliviaxu@szu.edu.cn

**Keywords:** phage display, metal-binding peptide, Cu(II), Aβ, Alzheimer’s disease

## Abstract

Copper (Cu) has been implicated in the progression of Alzheimer’s disease (AD), and aggregation of Cu and amyloid β peptide (Aβ) are considered key pathological features of AD. Metal chelators are considered to be potential therapeutic agents for AD because of their capacity to reduce metal ion-induced Aβ aggregation through the regulation of metal ion distribution. Here, we used phage display technology to screen, synthesize, and evaluate a novel Cu(II)-binding peptide that specifically blocked Cu-triggered Aβ aggregation. The Cu(II)-binding peptide (S-A-Q-I-A-P-H, PCu) identified from the phage display heptapeptide library was used to explore the mechanism of PCu inhibition of Cu^2+^-mediated Aβ aggregation and Aβ production. In vitro experiments revealed that PCu directly inhibited Cu^2+^-mediated Aβ aggregation and regulated copper levels to reduce biological toxicity. Furthermore, PCu reduced the production of Aβ by inhibiting Cu^2+^-induced BACE1 expression and improving Cu(II)-mediated cell oxidative damage. Cell culture experiments further demonstrated that PCu had relatively low toxicity. This Cu(II)-binding peptide that we have identified using phage display technology provides a potential therapeutic approach to prevent or treat AD.

## 1. Introduction

Alzheimer’s disease (AD) is a progressive neurodegenerative disease characterized by irreversible and progressive cognitive dysfunction and causes substantial medical and socioeconomic burden worldwide. AD has been hypothesized to be a protein misfolding disease caused by the accumulation of misfolded amyloid-β peptide (Aβ) and tau protein, which are the major pathological hallmarks of AD [1,2]. Aβ is produced by the sequential cleavage of β-amyloid precursor protein (APP) by two enzymes, β-secretase and γ-secretase [3]. Aβ readily self-assembles into a variety of aggregates called oligomers, fibrils, and mature amyloid fibers. Aβ1-40 and Aβ1-42 are the two most abundant forms of Aβ in the brain. The level of Aβ1-40 is approximately 10 times higher than that of Aβ1-42. Moreover, Aβ1-42 readily forms aggregates and has higher neurotoxicity than Aβ1-40. Aβ deposition gradually causes neuronal death and loss of cognitive function. Therefore, modulating the pathway of Aβ production and aggregation is critical for the pathogenesis of AD [4,5,6,7,8].

Increasing evidence suggests that the homeostasis of transition metal ions plays a pathogenic role in AD [9,10]. Accumulation of copper, zinc, and iron has been found in several regions of the AD brain, cerebrospinal fluid, and senile plaques [11,12,13]. Thus far, the underlying mechanism between copper accumulation and AD has not been completely understood. Clinical research has shown that copper levels in the brains of AD patients were significantly higher than those in the brains of normal people in the same age group, and the levels of copper in the brain accumulated with age [14,15]. Furthermore, it was shown that copper ions can directly bind to Aβ, promote the intermolecular cross-link of Aβ, and finally cause Aβ aggregation [16]. In addition, it has been proposed that copper ions can bind to both the APP and tau proteins [17]. The site-directed redox activity of copper can produce APP fragmentation and promote Aβ aggregation, therefore contributing to the formation of senile plaques [18]. Additionally, the increased level of redox copper produces toxic hydrogen peroxide and hydroxyl free radicals, which in turn destroy the integrity of the cell and cause neuronal damage [19,20,21,22,23,24].

Metal chelators are considered potential therapeutic agents for AD because of their capacity to reduce metal-induced Aβ aggregation and neurotoxicity by regulating the distribution of metal ions [25,26]. Several studies have demonstrated that copper chelating agents can reverse Aβ deposition and reduce neurotoxicity both in vitro and in the brains of AD model mice, which provides some validation for the hypothesis that copper ions are involved in the pathogenesis of AD [27,28]. The copper chelator clioquinol (CQ), which has an affinity for copper and zinc, can reduce aggregation of Aβ induced by metals in vitro [29,30]. Moreover, trientine can significantly reduce the APP/presenilin-1 (PS1) mouse brain senile plaques and reduce copper and zinc accumulation in plaques [31,32]. However, most chelating agents are chemical drugs, suggesting challenges in application and associated side effects, such as difficulty in passing through the blood-brain barrier and strong liver and/or kidney damage, respectively.

Recently, metal-binding peptides selected from phage display libraries as biocompatible metal chelators were shown to be potentially useful for the treatment of AD [33]. The most attractive advantage of phage display technology is that novel peptides for specific targets can be identified without previous knowledge of the interaction between the peptide and the target [34,35]. Furthermore, the binding specificity to the target can be regulated through repeated biopanning [36,37]. In our recent study, we successfully screened a specific Zn(II)-binding peptide based on phage display technology, and identified a Zn(II)-binding peptide that could inhibit Zn^2+^-mediated Aβ aggregation and neurotoxicity, therefore improving cognitive dysfunction in APP/PS1 transgenic mice. We provided a general pipeline for the development of metal chelating agents (for anti-metal-mediated Aβ aggregation) by binding the target metal ion with peptides based on a phage library [33].

In this study, we identified novel Cu(II)-binding peptides to develop physiologically compatible Cu^2+^ chelators and discussed their application prospects in metal-Aβ-mediated AD therapy. The Cu^2+^-chelating ability of the selected Cu(II)-binding peptide and inhibition of Cu^2+^-mediated Aβ aggregation-related neurotoxicity were confirmed. Our most promising specific chelator can be considered to be a potential regulator of copper homeostasis in neurodegenerative diseases.

## 2. Results and Discussion

### 2.1. Biopanning for Cu(II)-Binding Peptides and Peptide Sequence Analysis

**Figure 1:**
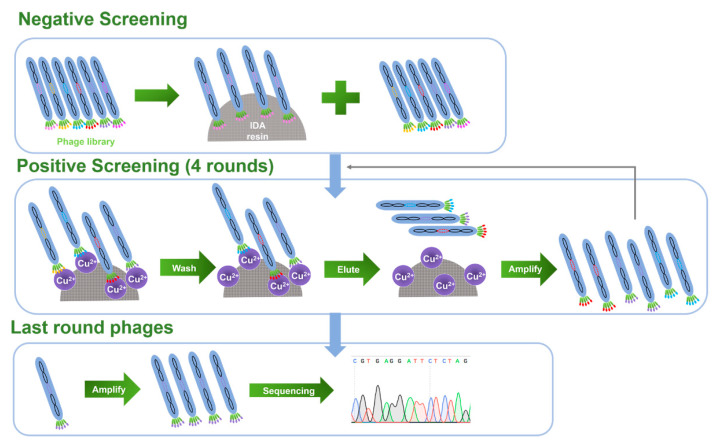
Schematic diagram illustrating the panning procedure.

To facilitate practical applications when screening for Cu(II)-binding peptides, a phage random heptapeptide library was selected due to the small molecular weight, good penetration, and low immunogenicity of the peptides. The diversity of the peptide library was ~10^9^, indicating that it contained ~10^9^ bacteriophage monoclonal antibodies. The input phage titer of each biopanning round was ~10^12^ pfu/mL, and the volume was 100 µL, which guaranteed that at least 100 copies of each phage clone was present, ensuring the effectiveness of the screening. In this experiment, a series of Cu(II)-binding heptapeptides were identified through a complete biopanning procedure, including one round of reverse biopanning against blank resin of IDA and four rounds of selection against immobilized Cu(II) resin (Figure 1). Reverse biopanning against blank IDA resin eliminated the phages that were bound non-specifically to the functional groups on IDA resins other than Cu(II). Following the reverse biopanning procedure, four rounds of selection against Cu(II) were conducted; during that process, phages were stripped off the immobilized Cu(II) resin using EDTA. The biopanning conditions were more stringent in the third round due to the reduced amount of Cu(II) loaded, which increased the binding affinity of the selected peptides. The increase in enrichment efficiency (Table S1) after each round of positive screening indicated that the phages bearing Cu(II)-binding peptides were efficiently enriched.

After the entire biopanning procedure, 15 Cu(II)-binding phage monoclonal peptides were randomly selected, and DNA sequences were successfully obtained for 12 of them. DNA sequences and the corresponding peptide sequences were analyzed as listed in Table 1, and no consensus motif was found in the 12 identified peptides. Meanwhile, we noticed that 6 of the 12 peptides contained histidine (H), which usually has a high affinity for metal ions, and most peptides contained hydroxyl (-OH)-containing amino acids (S, T, Y) that were connected by non-polar amino acids, which are also capable of metal binding [36,38]. Furthermore, peptide sequences containing a serine or threonine followed by a histidine residue have been confirmed in related studies on specific metal-binding peptides (Zn^2+^ [39], Cd^2+^ [40], Ni^2+^ [41,42]).

### 2.2. Selection of Candidate Peptide Binding to Cu(II)

**Figure 2:**
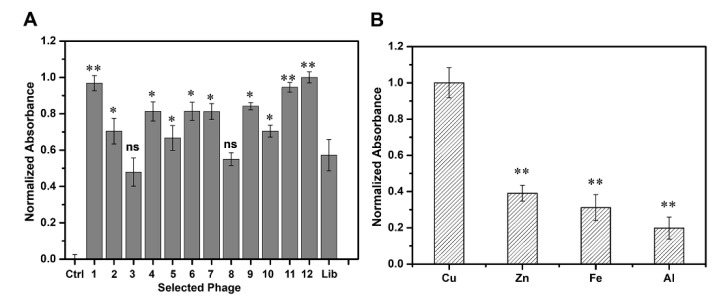
Binding affinity and selectivity of phage clones. (**A**) Comparison of the binding affinity to Cu(II) among the 12 selected phages. Binding affinity is represented by the normalized absorbance at 405 nm. Ctrl: Only Cu(II), Lib: Phage library. The data represent the mean ± S.E. of three independent experiments. ns = not significant; * *p* < 0.05; ** *p* < 0.01 compared to the Lib group. (**B**) Binding selectivity of P-12. Binding affinity toward different metals was represented by the normalized absorbance at 405 nm. The data represent the mean ± S.E. of three independent experiments. ** *p* < 0.01 compared to the Cu group.

We performed an ELISA assay to determine the binding affinity of the 12 selected peptides to Cu(II) (Figure 2A). Compared with the phage library, the clones bearing Cu(II)-binding peptides exhibited higher binding affinity to Cu(II), which confirmed that our biopanning procedure was effective with P-1, P-11, and P-12 exhibiting relatively high affinity. We then compared and analyzed the respective amino acid sequences and found that P-1 (VGYSGRD) and P-11 (GTQFFNK) did not contain histidine, whereas P-12 (SAQIAPH) contained a histidine at the C-terminal accompanied by a serine (containing hydroxyl) at the N-terminus, which may be more conducive in the coordination of the peptide association with copper ions [43,44]. Taken together, we finally chose P-12, which displayed SAQIAPH sequence as the candidate peptide for further study. BLAST analysis of the selected peptide on the National Center for Biotechnology Information database showed that there was no homologous sequence in the database, which indicated that the peptide was a novel peptide, and we named it PCu. The synthesized peptide (PCu) was identified using HPLC and mass spectrometry analysis (Supplementary Figure S1). To investigate whether the synthetic peptide PCu and its corresponding phage clone competed for the same binding site, we performed a competitive inhibition ELISA assay. The binding of the corresponding phage clones to Cu(II) was inhibited in a dose-dependent manner, demonstrating that PCu bound to Cu(II) by replacing the positive phage clones (Supplementary Figure S2). When the PCu concentration increased to 10 µM, the inhibition ratio reached 55% and was gradually saturated, which indicated that PCu and the corresponding phage clone P-12 were competing for the same binding site. The binding of clone P-12 to Cu(II) was indeed mediated by the PCu displayed on its surface. Additionally, due to the similarity of transition metal ions, it is possible that PCu could binds with other metal ions such as Zn(II), Fe(II) or et al. Therefore, we further investigated the affinity between PCu and other metal ions related to AD including Zn(II), Fe(III), and Al(III). As shown in Figure 2B, PCu bound with Cu(II) over other metals which indicated that PCu to be a metal-binding peptide with relative selectivity for target metals.

### 2.3. Inhibition of Cu^2+^-Mediated Aβ1-42 Aggregation by PCu In Vitro

**Figure 3:**
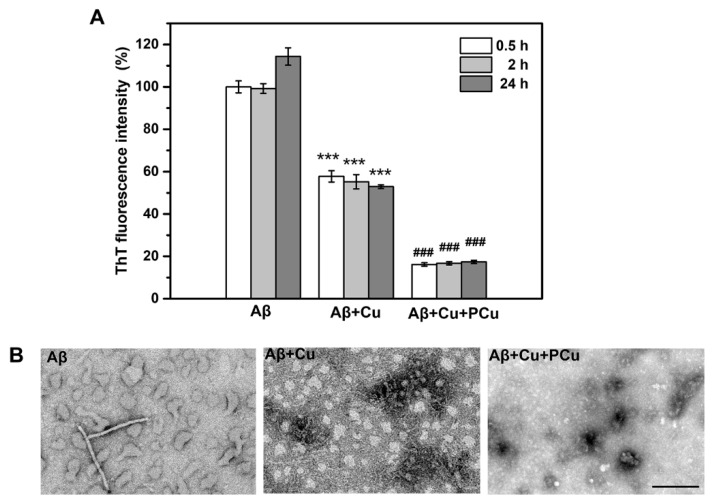
PCu inhibits Cu^2+^-induced aggregation of Aβ in vitro. (**A**) ThT fluorescence was measured at 0.5, 2 and 24 h after incubating Aβ1-42 with 25 μM Cu(II) and 25 μM PCu. (**B**) The corresponding TEM images of Aβ aggregation form under different solution conditions. Scale bar: 200 nm. The data represented the mean ± S.E. of three independent experiments. *** *p* < 0.001 compared to the Aβ group, ### *p* < 0.001 compared to the Cu^2+^-induced group.

A key etiology of AD is the enrichment of Cu^2+^ in Aβ aggregates, which can rapidly induce oligomerization of Aβ peptides in the presence of Cu^2+^ [45]. It has been demonstrated that the affinity of Cu^2+^ for Aβ1-42 is higher than that for Aβ1-40, indicating that Cu^2+^ can easily promote the aggregation of Aβ1-42 oligomerization [46]. Therefore, in this study, Aβ1-42-Cu(II) aggregation was used to characterize and assess the ability of PCu to inhibit Aβ1-42 aggregation. The ability of PCu to inhibit the aggregation of Aβ1-42-Cu(II) was monitored using a ThT fluorescence assay at 0.5, 2 and 24 h. As shown in Figure 3A, PCu inhibited the formation of Aβ1-42-Cu(II) aggregates by about 60% at 2 h, which remained at 60% after 24 h, suggesting that as long as PCu was present, the fluorescence intensity of Aβ1-42 aggregates could be maintained at a low level. In addition, compared with Aβ1-42 alone, the addition of Cu(II) reduced the ThT fluorescence level, suggesting that Cu(II) triggered the formation of partial Aβ1-42 oligomers because ThT selectively binds to amyloid fibrils but does not interact with unstructured Aβ monomers and oligomers [47,48]. Therefore, the ThT fluorescence intensity was not as high as that in the presence of Aβ1-42 alone, which is consistent with previous studies [49,50]. In addition, the corresponding TEM analysis was performed to observe the morphologies of the Aβ-Cu(II) aggregates (Figure 3B). After 24 h, Aβ1-42 appeared shorter fibrils, and aggregated into amorphous aggregates that were stacked together under Cu^2+^ treatment. At the same time, the presence of oligomers could be observed incubated with Cu^2+^. In contrast, in the presence of PCu, we could observe only a few small amorphous and granular aggregates indicating that PCu can inhibit Cu^2+^ mediated Aβ aggregation in vitro.

### 2.4. PCu Attenuated Cu^2+^-Mediated Cell Damage and Oxidative Stress

**Figure 4:**
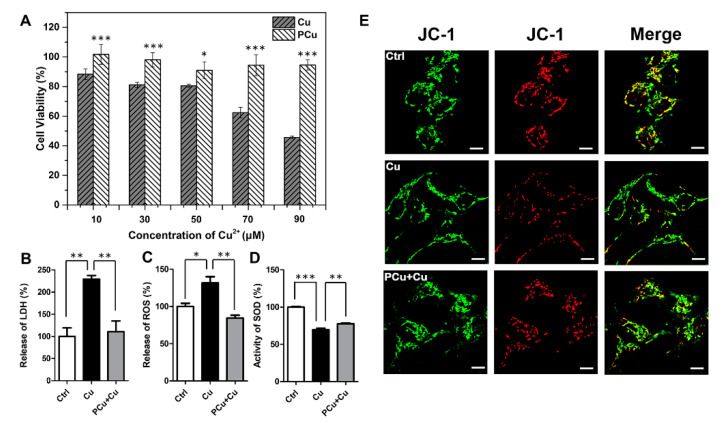
PCu attenuates Cu^2+^-mediated cell damage and oxidative stress. (**A**) N2a-sw cells incubated with different concentrations of Cu(II) or 100 μM PCu for 12 h to detect cytotoxicity in N2a-sw cells using MTT assay. (**B**) Release of LDH (**C**) The production of ROS. (**D**) SOD activity in N2a-sw cells. (**E**) Representative photomicrographs of JC-1 fluorescent staining in the N2a-sw cells. Scale bar: 10 μm. The data represent the mean ± S.E. of three independent experiments. * *p* < 0.05; ** *p* < 0.01; *** *p* < 0.001 compared to the Cu^2+^-damaged group.

N2a-sw cells were used as an AD model in vitro to further examine the biocompatibility of PCu and its efficiency in ameliorating neurotoxicity induced by Cu^2+^. Within the scope of the experimental conditions (Supplementary Figure S3), PCu showed almost no toxicity to cells even with increased incubation times and PCu concentration. In fact, cells treated with PCu displayed a certain degree of proliferation, indicating good biocompatibility of PCu. Furthermore, the ability of PCu to rescue N2a-sw cells from Cu^2+^-mediated toxicity was evaluated using the MTT assay. As shown in Figure 4A, after incubation with different concentrations of Cu^2+^ (10, 30, 50, 70, and 90 μM) for 12 h, cell viability gradually decreased. Specifically, viability decreased to below 50% at 90 μM Cu^2+^. However, when cells were co-incubated with 100 μM PCu, the cell viability rates were maintained at ~100% even with a Cu^2+^ concentration up to 90 μM. Then, we performed lactate dehydrogenase (LDH) detection assay and treated N2a-sw cells as described above. As shown in Figure 4B, compared to Cu^2+^-treated cells, the levels of LDH were dramatically decreased in the presence of PCu.

Furthermore, mitochondrial membrane potential (MMP) is a prerequisite for maintaining mitochondrial oxidative phosphorylation to produce adenosine triphosphate. The stability of MMP is conducive to maintaining the normal physiological functions of cells. Apoptosis and oxidative stress are closely related to the stability of MMP [51]. Therefore, we also investigated the effect of PCu on the membrane stability of mitochondria by JC-1 fluorescent staining. JC-1 is widely used to detect MMP due to the discrimination of energized and deenergized mitochondria [52,53]. JC-1 fluorescent staining exhibits potential-dependent accumulation in mitochondria: when mitochondria in higher membrane potential, normally green fluorescence will form red fluorescent aggregates. Therefore, the change of color directly reflects the function of MMP. As shown in Figure 4E, the red fluorescence decreased significantly after Cu^2+^ treatment, and the red fluorescence recovered when PCu incubated with Cu^2+^ which can also be observed more clearly in the merge images. The co-localized yellow color exhibited the change of MMP indicating that PCu can stabilize Cu^2+^-meditated MMP reduction. Taken together, these results highlight the potential of PCu to rescue N2a-sw cells from Cu^2+^-mediated cytotoxicity.

Redox active metal ions (such as copper) can catalyze the production of reactive oxygen species (ROS). The generated ROS may cause oxidative damage to Aβ itself and surrounding molecules (proteins, lipids, etc.) [54]. Free or loosely bound copper molecules are effective catalysts for ROS production. They can be reduced to Cu(I) by physiologically relevant reducing agents (such as glutathione or ascorbate), and react with hydrogen peroxide to form superoxide and hydroxyl radicals. Therefore, an increase in the concentration of free or loosely bound copper molecules can usually catalyze the production of ROS. When the production of ROS is above normal levels, cells respond to oxidative stress by activating cell death program [55,56]. Superoxide dismutase (SOD) content is directly proportional to the ability of scavenging free radicals. Therefore, we detected the production of ROS and SOD activity in N2a-sw cells (Figure 4C,D). Compared with Cu^2+^-treated cells, PCu significantly reduced Cu^2+^-induced ROS production and increased SOD activity. These results indirectly indicate that PCu can protect N2a-sw cells from Cu^2+^-mediated cell damage through its antioxidant properties.

### 2.5. PCu Inhibited Cu^2+^-Mediated Aβ Deposition In Vitro

**Figure 5:**
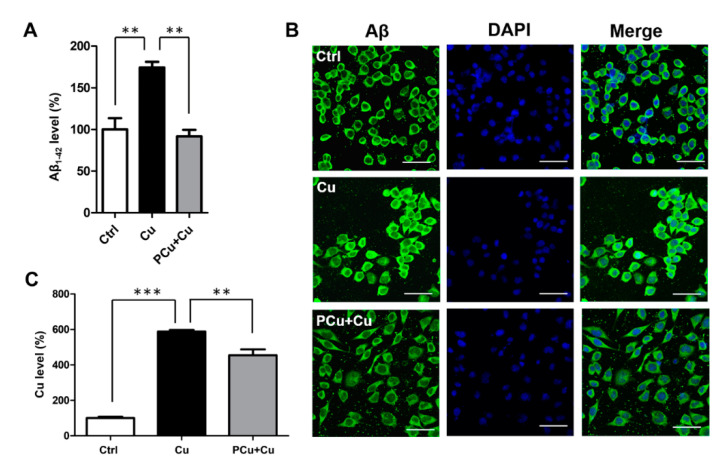
PCu inhibits Cu^2+^-mediated Aβ deposition in N2a-sw cells. N2a-sw cells were treated with 50 μM of Cu(II) or co-incubated with 100 μM PCu for 12 h. (**A**) Extracellular Aβ secretion is measured using ELISA. (**B**) Representative photomicrographs of Aβ fluorescence in the N2a-sw cells. Scale bar: 20 μm. (**C**) Detection of the intracellular copper content using ICP-MS. The data represent the mean ± S.E. of 3 independent experiments. ** *p* < 0.01; *** *p* < 0.001 compared to the Cu^2+^-damaged group.

Quantification of Aβ production demonstrated that compared with the control group, Cu^2+^ treatment significantly increased the Aβ secretion in N2a-sw cells, while the addition of PCu significantly inhibited Cu^2+^-induced Aβ production (Figure 5A). Consistent with this result, Aβ immunofluorescence staining also showed that intracellular Aβ accumulation promoted by Cu^2+^ decreased after treatment with PCu (Figure 5B). Metal analysis using ICP-MS showed that there was a significant increase in intracellular copper content after the addition of extra copper, and PCu treatment decreased the intracellular level of copper in cells (Figure 5C). Taken together, these results indicate that PCu could inhibit the production of Aβ in N2a-sw by binding copper.

**Figure 6:**
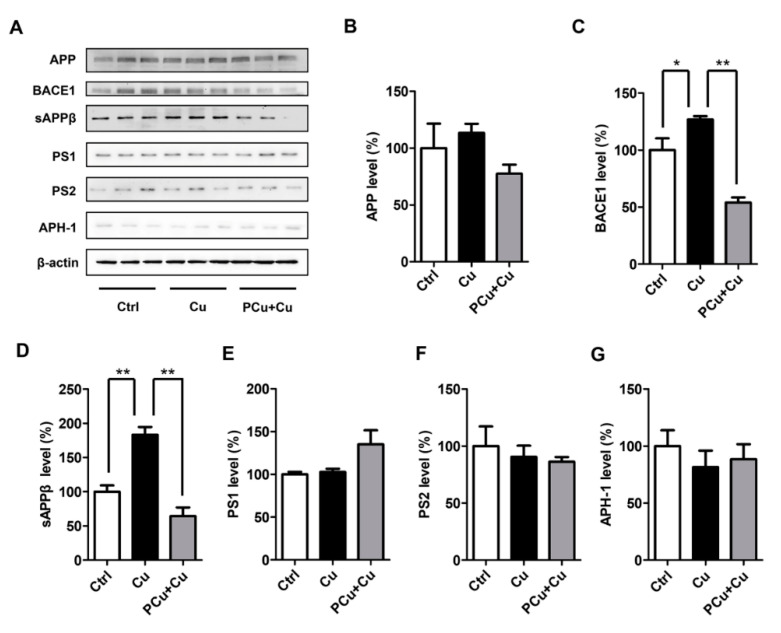
The effect of PCu and Cu(II) on the expression of cleavage enzymes of APP and intermediates in N2a-sw cells. PCu inhibited Cu^2+^-mediated Aβ deposition in N2a-sw cells. N2a-sw cells were treated with 50 μM of Cu(II) or co-incubated with 100 μM PCu for 12 h. (**A**) The expression of APP, BACE1, sAPPβ, PS1, PS2 and APH-1 detected using Western Blot. (**B**–**G**) Quantitative analysis of the expression of APP, BACE1, sAPPβ, PS1, PS2 and APH-1. The data represent the mean ± S.E. of three independent experiments. * *p* < 0.05, ** *p* < 0.01 compared to the Cu^2+^-damaged group.

Copper ions have also been shown to directly affect the production of Aβ through the Aβ synthesis pathway (promoting the amyloid pathway of APP), therefore intensifying Aβ deposition [57,58]. Therefore, to clarify the mechanism by which PCu reduced Aβ production, we also examined the levels of APP and the enzymes involved in APP cleavage of the amyloid pathway using Western Blot analysis (Figure 6). No significant differences in the protein levels of full-length APP (fl-APP) and γ-secretase were observed in either group. Compared to the Cu^2+^-induced group, PCu significantly decreased the Cu^2+^-induced BACE1 protein and intermediate products of APPβ expression levels, indicating that PCu could reduce the production and deposition of Aβ by inhibiting the secretase activity of the amyloid pathway.

APP has a copper-binding site in its N-terminal extracellular domain [59,60], and may be involved in the regulation of copper oxide in the brain [61,62]. The combination of copper and APP seems to promote the dimerization of APP. This may further promote the localization of BACE1 in lipid rafts, where BACE1 is concentrated, therefore promoting its shearing and elongation, and increasing the production of Aβ. In addition, copper-induced oxidative stress may be related to BACE1 expression in neurons. Recent studies have shown that BACE1 has a copper-binding site in its cytoplasmic domain and that BACE1 interacts with copper clad steel (CCS), which is an important protein that transports copper to SOD1 for activation. Because BACE1 competes with SOD1 for interaction with CCS, the up-regulation of BACE1 results in decreased SOD1 activity in cells [63,64]. Our results showed that copper up-regulated BACE1 in N2a-sw cells, and the addition of PCu significantly inhibited Cu^2+^-induced BACE1 expression and Aβ secretion. PCu also improved Cu^2+^-mediated oxidative damage in cells. In short, PCu prevented binding of metal ions to Aβ and inhibited Aβ aggregation.

### 2.6. PCu Enters the Brain after Intranasal Administration

**Figure 7:**
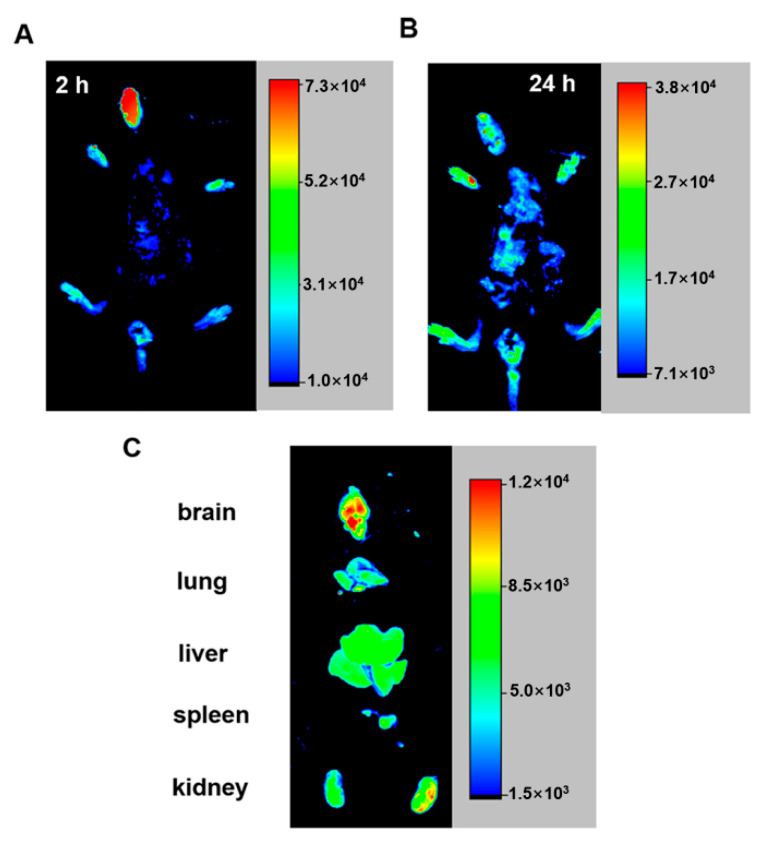
Optical in vivo imaging of the FITC-PCu administered via mouse vein. (**A**,**B**) Images are taken 2 and 24 h after FITC-PCu administration. (**C**) Ex-vivo imaging of FITC-PCu in main organs 24 h after FITC-PCu administration.

Currently, intranasal administration is the recommended method for intracerebral administration to avoid the necessity to penetrate the blood-brain barrier [65,66,67]. Therefore, in this study, FITC-labeled PCu was administered to mice through the nasal cavity. We selected 2 h (short-term intake) and 24 h (long-term intake) to observe the distribution of PCu in nude mice. After 24 h, the main organs (brain, lung, liver, kidney, and spleen) of the mice were removed, and the distribution of PCu in these organs was analyzed using a small-animal live imaging instrument. As shown in Figure 7A, PCu quickly accumulated in the mouse brain and gradually weakened over time. Except for the brain, fluorescent signals were mainly observed in the liver and kidneys of mice (Figure 7B). The kidney is the main excretion organ of mice, and peptides are easily soluble in water, which may be the main metabolic pathway of PCu. Collectively, we have shown the potential of PCu for the treatment of metal-mediated AD and that it may be suitable for the treatment of brain diseases, although further research is needed.

## 3. Materials and Methods

The materials used in this study and detailed biopanning procedures are described in the Supporting Information.

### 3.1. Thioflavin T Assays

First, dissolved the Aβ1-42 lyophilized powder in HFIP solution to a final concentration of 1.0 mg/mL, then incubated for 2 h at 4 °C, sonicated it in an ice bath for 2 min, and centrifuged the resulting solution (4 °C, 12,000 rpm, 30 min) to remove pre-existing aggregates [67]. Collected 75% of the top supernatant to freeze-dry storing at −20 °C for use. For the inhibition of Cu^2+^-mediated Aβ1-42 aggregation, Aβ1-42 was dissolved in 100 μL DMSO, which was fully dissolved by ultrasonic treatment and diluted with double-distilled water to a final concentration of 50 μM. The mixture of the Aβ1-42 (25 μM) with Cu^2+^ (25 μM) with or without the presence of PCu (25 μM) was incubated at 37 °C, and 100 μL of each group was taken out at different times. Then, the above solution was mixed with ThT (50 μM) in a plate, and the fluorescence (Ex/Em = 440/480 nm) was detected on a fluorescence microplate reader at (Synergy H1, BioTek, Winooski, VT, USA).

### 3.2. Transmission Electron Microscopy

The samples from the ThT assay (after incubated for 24 h) were respectively placed on the 400-mesh copper grids covered with carbon film for 5 min and allowed to dry. Then negatively stained with 2% phosphotungstic acid for 30 s and observed in a JEM-1400Plus (JEOL, Tokyo, Japan) operating at 200 kV.

### 3.3. Cells

Mouse neuroblastoma (N)2a cells stably overexpressing APP Swedish mutation (N2a-sw) were donated by Professor Huaxi Xu (College of Medicine, Xiamen University) [66]. The cells were grown on 6-cm tissue culture dishes in 3 mL Dulbecco’s Modified Eagle Medium (DMEM) supplemented with 10% fetal bovine serum (FBS) and 200 µg/mL G418 (Sigma-Aldrich). After undergoing culture in an FBS-free medium for an additional 6 h, the cells were treated with different formulations after growing in a serum-free medium for an additional 4 h in sets of experiments. The control groups were incubated in culture medium alone.

An MTT assay was conducted to evaluate the effect of Cu(II) and PCu on the viability of N2a-sw cells according to the literature procedure [68]. In brief, N2a-sw cells were seeded in 96-well plates, in each separate experiment, cells were treated with different concentrations of PCu (0.1, 1, 10, 100, 500 μM) for 6 h, 12 h or 24 h, or different concentrations of Cu(II) (10, 30, 50, 70, 100 μM) with or without the presence of PCu (100 μM) for 12 at 37 °C. Subsequently, 20 μL of MTT (Sigma-Aldrich, Burlington, MA, USA) was added to each well and further incubated for 4 h. The absorbance was measured at 490 nm in a microplate reader (Bio-Rad, Hercules, CA, USA). The detection of lactate dehydrogenase (LDH) release, reactive oxygen species (ROS), and superoxide dismutase (SOD) (commercial kits, Jiancheng Biology, Nanjing, China) of the N2a-sw cells were carried out according to the manufacturer’s instructions.

### 3.4. ELISA-Based Measurement of Aβ Level

The Aβ1-42 level was determined using Wako Human β Amyloid (142) ELISA High-Sensitivity Kit (296-64401, Wako, Richmond, VA USA) according to the manufacturers’ instructions as previously described [68]. Total protein level was measured at 450 nm recorded with an ultra-violet spectrophotometer.

### 3.5. Immunofluorescence Staining

Aβ immunofluorescence assay was generated as previously described [33]. Briefly, after treatment, the N2a-sw cells were fixed with 4% paraformaldehyde for 30 min. Cells were then incubated for 2 min with 0.1% Triton X-100. The sections were incubated in buffer containing 5% goat serum/PBS for 30 min at room temperature, and then incubated overnight at 4 °C with a mouse antibody against Aβ. After washing three times with PBS, sections were incubated with Alexa Fluor^®^ 488-conjugated goat anti-mouse IgG (1:1000; Invitrogen, Carlsbad, CA, USA) in the dark for 2 h at room temperature. Finally, cells were incubated in DAPI for 5 min. Images were taken under a confocal microscope (Leica Microsystems Ltd., Wetzlar, Germany).

JC–1 (Sigma-Aldrich, Burlington, MA, USA), a fluorescent lipophilic carbocyanine dye, were used to measure mitochondrial membrane potential. Briefly, after corresponding treatment, culture media were removed from the N2a-sw cells and replaced with JC-1 working solution (final concentration 2 μM). Then, cells were incubated in a dark place at 37 °C for 15–20 min. After washing two times with PBS, added appropriate amount of PBS to the cells, images were taken under a confocal microscope (Green fluorescence: Ex/Em = 510/527 nm, Red fluorescence: Ex/Em = 585/590 nm).

### 3.6. Copper Analysis with Inductively Coupled Plasma Mass Spectrometry (ICP-MS)

To measure copper levels of cells, samples were digested in 90% HNO_3_ at 105 °C for 30 min. Then, samples were diluted to the appropriate multiple before detection using a 7500a-ICP-MS (Aglient Technologies Inc., Santa Clara, CA, USA). The data acquisition mode (spectral analysis) was set to ^63^Cu.

### 3.7. Western Blot Analysis

After treatment, cells were lysed by RIPA lysis buffer and electrophoresed on SDS-polyacrylamide gel with a loading volume of 1 µg/µL. Proteins (10 μg) were separated onto 4–12% SDS-polyacrylamide gels and transferred onto polyvinylidene fluoride (PVDF) membranes. Then membranes were incubated in 5% BSA solution at room temperature for 1 h. Subsequently, the membranes were respectively incubated with primary antibodies: rabbit-anti-APP C-terminal (A8717, Sigma-Aldrich, Burlington, MA, USA), rabbit-anti-BACE1 (ab108394, Abcam, Cambridge, UK), rabbit-anti-Presenilin 1 (5643, Cell Signaling Technology, Danvers, MA, USA), rabbit-anti-Presenilin 2 (9979, Cell Signaling Technology, Danvers, MA, USA), mouse-anti-Human sAPPβ-sw (10,321, IBL, Minneapolis, MN, USA), rabbit-anti-APH1 (AB9214, Millipore, Burlington, MA, USA), mouse-anti-β-Actin (A1987, Sigma-Aldrich, Burlington, MA, USA) overnight at 4 °C. Membranes were washed with TBST and subsequently incubated with the horseradish peroxidase-conjugated anti-rabbit or mouse secondary antibodies (1:5000) for 1 h at room temperature. The bands were processed with ECL kit (WBKLS0500, Millipore, Burlington, MA, USA) and observed in the chemiluminescence imaging system (ChemiOoc XRS+, Bio-Rad, Hercules, CA, USA). Protein intensities were semi-quantitatively analyzed using the NIH ImageJ software [66,69].

### 3.8. Animal Imaging Analysis

C57BL/6 mice (Beijing HuaFuKang Bioscience Co., Ltd., Beijing, China) were administered to through the nasal cavity with FITC-labeled PCu (2 mg/kg) or PBS solution [2]. The mice were then anesthetized by intraperitoneal injection of 4% chloral hydrate at 2 h and 24 h, and animals were humanely killed for brain, lung, liver, kidney, and spleen isolation. All images were captured and analyzed using Carestream FX MS PRO (Bruker, Billerica, MA, USA). All animal procedures were conducted in accordance with the Guidelines for Care and Use of Laboratory Animals of Northeastern University and approved by the Animal Ethics Committee of College of Life and Health Sciences of Northeastern University.

### 3.9. Statistical Analysis

All data are presented as the mean ± SEM. All statistical values are from at least three independent experiments. Comparisons were performed using one-way analysis of variance (ANOVA), followed by Fisher’s protected least significant difference (PLSD) multiple comparison tests or two-tailed Student’s t-tests. Results with *p* < 0.05 were considered significant.

## 4. Conclusions

In the current study, a novel Cu(II)-binding peptide (S-A-Q-I-A-P-H) was identified from a phage display heptapeptide library as a copper ligand for the possible treatment of metal-mediated AD. PCu not only chelates Cu^2+^, but also inhibits Cu^2+^-induced Aβ1-42 aggregation in vitro, and attenuates Cu^2+^-mediated oxidative stress in N2a-sw cells. Notably, the ability of PCu to rescue N2a-sw cells from Cu-Aβ1-42-induced toxicity makes it a promising candidate for further evaluation. Furthermore, PCu inhibited the levels of β-secretase BACE1 and sAPPβ to inhibit the production of Aβ aggregates. Hence, PCu could be an important metal-directed ligand in metal-mediated AD.

## Figures and Tables

**Table 1:** Peptide sequences of the selected Cu(II)-binding phage.

No.	Peptide Sequence	Frequencies of Histidine and -OH Containing Amino Acid
P-1	VGYSGRD	2
P-2	GYWNKFD	1
P-3	HGSGVHA	3
P-4	VIPQEIF	0
P-5	EHHRSHL	4
P-6	YMNDRMY	2
P-7	APGGHSS	3
P-8	TGLIGQK	1
P-9	DKSHVGL	2
P-10	HPIKHLR	2
P-11	GTQFFNK	1
P-12 ^a^	SAQIAPH ^a^	2

^a^ The phages bearing peptide P-12 bound to Cu^2+^ with the highest affinity.

## Data Availability

The data that support the findings of this study are available from the corresponding author upon reasonable request.

## Supplementary Materials

The following are available online at https://www.mdpi.com/article/10.3390/ijms22136842/s1, The materials used in this study and detailed biopanning procedures, Figure S1: Analysis of the synthetic PCu and Fitc-PCu, Figure S2: Concentration-dependent inhibition of PCu to phage clone, Figure S3: The effect of PCu on cell activity, Table S1: The phage titer results at each stage of each biopanning procedure.
