# Supplementary material for: A Novel Cu(II)-Binding Peptide Identified by Phage Display Inhibits Cu2+-Mediated Aβ Aggregation
Source: Int J Mol Sci. 2021 Jun 25;22(13):6842. doi: 10.3390/ijms22136842 (PMC8269028; doi:10.3390/ijms22136842)
Supplement: Supplementary file 1 [file ijms-22-06842-s001.zip › ijms-1210186-supplementary.pdf]

Supporting Information

A Novel Cu(II)-Binding Peptide Identified by Phage Display Inhibits Cu^{2+}-Mediated Aβ Aggregation

Xiaoyu Zhang[a,c,#], Xiancheng Zhang[a,c,#], Manli Zhong[a], Pu Zhao[a], Chuang Guo[a], You Li[a], He Xu[a], Tao Wang[a], Jiazhen Wu[a], Huiling Gao[a,b,*]

[a]College of Life and Health Sciences, Northeastern University, Shenyang, China

[b]Key Laboratory of Data Analytics and Optimization for Smart Industry, Northeastern University, Ministry of Education, China

[c]Key Laboratory of Separation Science for Analytical Chemistry, Dalian Institute of Chemical Physics, Chinese Academy of Sciences, China

# These authors contributed equally to this work.

*Corresponding author

Huiling Gao

College of Life and Health Sciences, Northeastern University, No. 3-11, Wenhua Rd., Shenyang 110819, China

E-mail: gaohuiling@mail.neu.edu.cn; Phone/Fax: +86-024-83656109.

1. Experimental details

1.1 Materials and chemicals

The peptide PCu was synthesized by China Peptides Co., Ltd (Suzhou, China) and purified by high performance liquid chromatography with a purity of >95%. The peptide PCu was labeled with FITC at the N-terminus. A phage display heptapeptide library and Escherichia coli ER2738 host cells were purchased from (New England Biolabs, Ipswich, MA, USA). N2a-sw cell lines were kindly provided from Prof. Hua-Xi Xu (Xiamen University). A dehydrogenase (LDH) release assay kit, reactive oxygen species (ROS) assay kit, and superoxide dismutase (SOD) assay kit were purchased from Jiancheng Institute of Biological Engineering (Nanjing China).

1.2 The preparation of Cu-chelating resin

Cu(II) immobilized resin was prepared by replacing the preloaded Ni(II) by Cu(II) on the Ni-IDA-Sefinose resin (Bio Basic Inc., Canada), as described previously [1,2]. In brief, resin was first washed with EDTA solution (0.5 mol L^{-1}, pH 8.0) to strip Ni(II) off the Ni-IDA-Sefinose resin. Then, the resin beads (blue-green) were washed three times with 0.05% Tween-20 in TBS (TBST) solution until they became colorless. To prepare Cu(II) immobilized resin, 1 mL of the metal-free resin suspension was incubated with 5 mL of Cu(II) solution ($CuSO_4$, 0.01 mol L^{-1}) overnight with gently shaking. The resin was rinsed six times with TBST solution and then stored at 4 ℃ for future use. Other metal-chelating resins were prepared by a similar method, except that the metal salts were replaced by $Fe(NO_3)_3 \cdot 9H_2O$, $ZnSO_4 \cdot 7H_2O$, and $Al_2(SO_4)_3 \cdot 18H_2O$.

1.3 Phage display biopanning procedures for screening the Cu(II)-binding peptide

The purpose of phage biopanning is to obtain peptides specifically binding to Cu(II). In order to eliminate the phages bound to the resin, the IDA resin was first subjected to reverse biopanning followed by Cu(II) affinity biopanning. In brief, 100 μL of the phage solution (~2 $\times 10^{12}$ plaque forming units, PFUS) in the NEB original peptide library was dissolved in 900 μL of TBST solution and added to 100 μL of IDA resin,

followed by gently shaking for 25 min at 25 ℃. The supernatant was extracted and further infected with E. coli ER2738 cell culture for amplification according to the manufacturer’s instructions. Then affinity screening was carried out for screening Cu(II)-binding peptide. 100 μL of the phage solution (~0.8×10^{12} virions) from the reverse screening amplification was dissolved in 900 μL of TBST solution, and added to 100 μL of Cu(II) chelating resin, followed by gently shaking for 25 min at 25 ℃. The supernatant was discarded, and the resin was washed twice with 1 mL of TBST solution to remove unbound phages. Finally, 1 mL of EDTA (0.5 M, pH 8.0) was added, and the eluate was collected by shaking at 200 rpm at 25 ℃ for 10 min. The eluate was centrifuged with a 100 KD centrifugal filter device (Millipore, U.S.) at 4 ℃, 5000 rpm, 15 min to remove Cu(II). The remaining phages are amplified and subjected to the next round of screening. Besides, to obtain high affinity phages, 4 rounds of biopanning were conducted. In the fourth round of biopanning, Cu(II) loaded on the resin beads was reduced by washing with citrate buffer solution (pH 4.4) to improve the biopanning affinity [2-4].

1.4 DNA sequence analysis

After completing all biopanning procedures, 15 well-isolated phages were randomly selected for DNA sequencing. Phage DNA was extracted by using M13 single-stranded DNA extraction kit (BioTeke, China) and DNA sequencing was entrusted to Shanghai Sangon Biotech Corporation (Shanghai, China).

1.5 Enzyme-linked immunosorbent assay

Enzyme-linked immunosorbent assay (ELISA) was used to evaluate the affinity and specificity of phage clones toward Cu(II). 100 μL individual phage clones were mixed with 1 mL of TBS, added to 200 μL of Cu(II) resin and incubated at 25 ℃ and 150 rpm for 1 h. Then the mixture was washed 3 times with TBST, and 1 mL (HRP)-conjugated anti-M13 antibody (GE healthcare, U.S.) (1:5000, diluted in TBST) was added at 37 ℃ for 1 h, followed by 3-time washing-step with TBST. Finally, 400 μL of substrate solution (220 mg L^{-1} ABTS and 0.17% H_2O_2 (30%) diluted in 50 mM sodium citrate

(pH 4.0) was added and allowed to incubate in the dark for 1 h for the color reaction to develop. Absorbance was recorded at 405 nm with an ultraviolet spectrophotometer (UV 1800 Pharma Spec, Japan). In additional, the ELISA was also applied to other metal-chelating resins.

1.6 Competitive inhibition assay

The competitive inhibition assay was used to verify the competitive binding effect between the phage monoclonal and its displayed peptide. In brief, PCu was added to Cu(II) resins (10 μM Cu^{2+}) at various concentrations (0.1, 1, 10, 100, 500 μM), and incubated for 2 h at room temperature. Next, 1.0×10^{11} pfu mL-1 PCu corresponding phages were added and incubated with gently shaking for 2 h at room temperature, followed by 3-time washing-step with TBST. (HRP)-conjugated anti-M13 antibody was added, and the absorbance was measured as ELISA assay mentioned above.

.

2. Supplemental figures

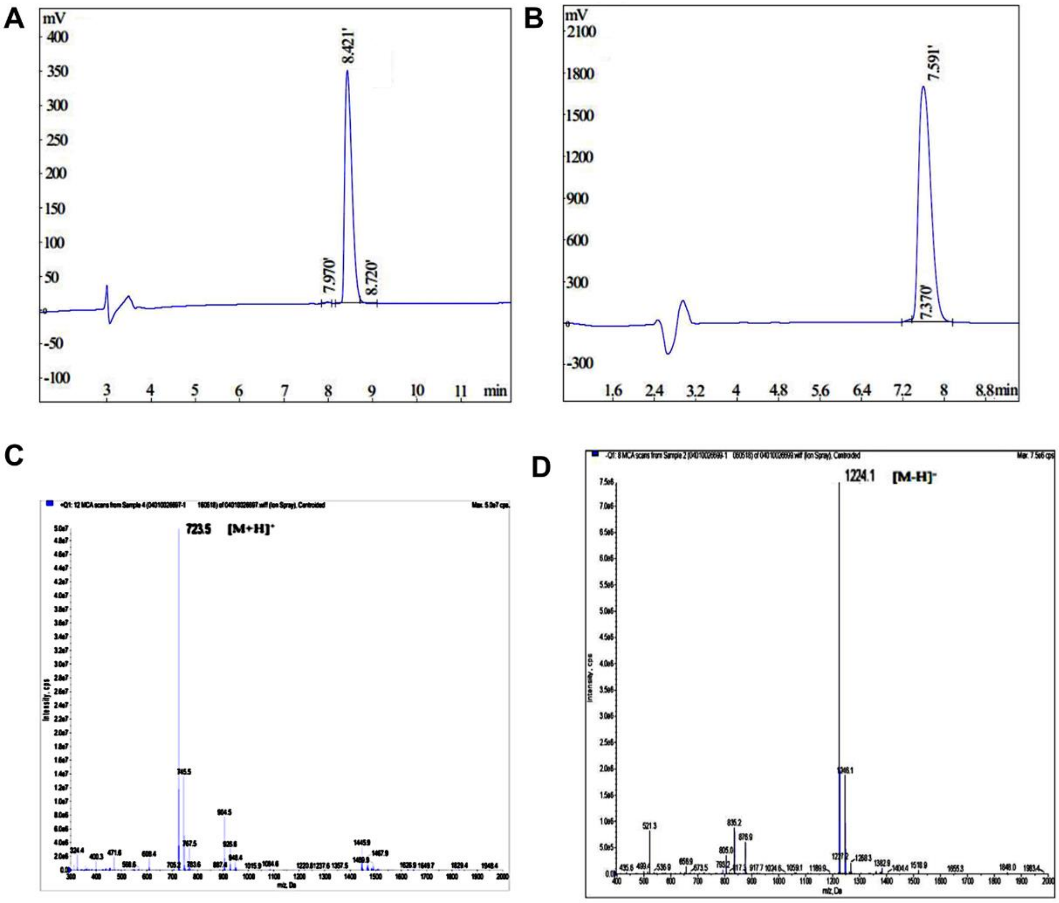

Figure S1. Analysis of the synthetic PCu and Fitc-PCu. (A-B) HPLC chromatogram. **(C-D)** Electrospray ionization mass spectrum.

The result of HPLC analysis showed that the purity of PCu and Fitc-PCu were greater than 95%. The results of mass spectrometry analysis showed that the molecular weights of synthesized PCu and Fitc-PCu were consistent with the theoretical values.

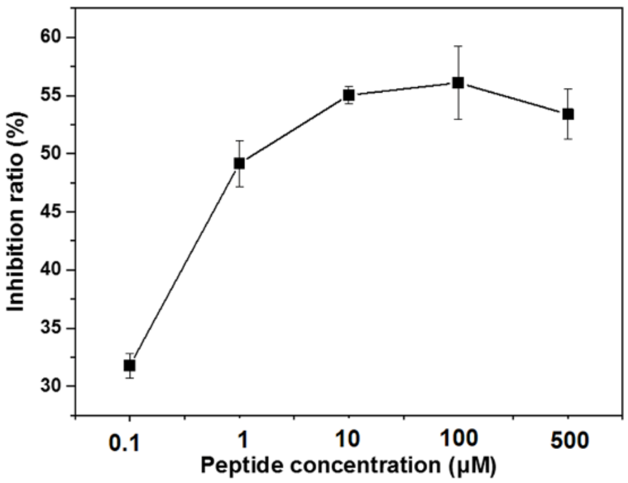

Figure S2. Concentration-dependent inhibition of PCu to phage clone.

PCu and the corresponding phage clone P-12 were competing for the same binding site indicating that the binding of clone P-12 to Cu(II) was mediated by PCu displayed on its surface.

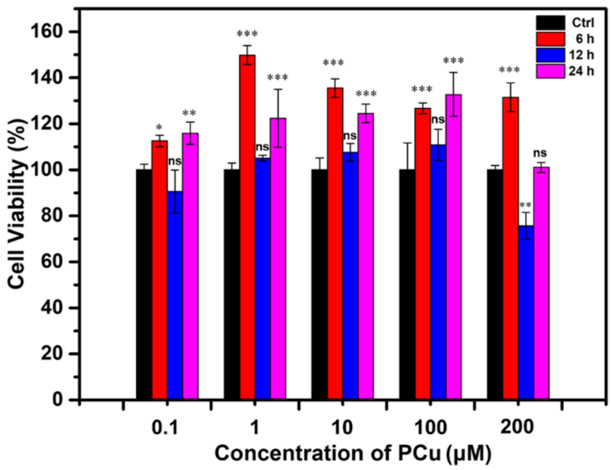

Figure S3. The effect of PCu on cell activity. Different concentrations of PCu (0.1, 1, 10, 100 and 500 μM) at different times (6, 12, and 24 h) on the survival rate of N2a-sw cells analyzed by MTT assay. The data represent the mean ± S.E. of 3 independent experiments. ns = not significant; * $p < 0.05$; ** p < 0.01; ***, $p < 0.001$ compared to the control group.

Within the scope of the experimental conditions, as the incubation time increased and the PCu concentration increased, PCu showed almost no toxicity to cells, and might even have a certain degree of promotion of cell proliferation, indicating that PCu has a good biocompatibility.

3. Supplemental tables

Table S1. The phage titer results at each stage of each biopanning procedure.

Biopanning steps		Phage Inputs 2.0×10^{12}			
		NO.	Recovered phage titer/ pfu mL^{-1}	Amplified for next round/ pfu mL^{-1}	Recov.*
Phage heptapeptide library	Negative screening against IDA	1st	7.6×10^{11}	—	—
	Positive screening against Cu(II)	1st	1.1×10^{8}	2.6×10^{12}	8.7×10^{-4}
		2ed	5.3×10^{9}	4.0×10^{12}	1.2×10^{-2}
		3rd	2.6×10^{10}	3.0×10^{12}	3.9×10^{-2}
		4th	1.1×10^{11}	—	**0.22**

Recovery = output/input = virions of supernatant/virions of amplified phage from last round.

4. References

1. Yang, T.; Zhang, X.Y.; Zhang, X.X.; Chen, M.L.; Wang, J.H. Chromium(III) Binding Phage Screening for the Selective Adsorption of Cr(III) and Chromium Speciation. *ACS Appl Mater Interfaces* **2015**, *7*, 21287-21294, doi:10.1021/acsami.5b05606.
2. Zhang, X.; Zhong, M.; Zhao, P.; Zhang, X.; Li, Y.; Wang, X.; Sun, J.; Lan, W.; Sun, H.; Wang, Z.; et al. Screening a specific Zn(ii)-binding peptide for improving the cognitive decline of Alzheimer's disease in APP/PS1 transgenic mice by inhibiting Zn(2+)-mediated amyloid protein aggregation and neurotoxicity. *Biomater Sci* **2019**, *7*, 5197-5210, doi:10.1039/c9bm00676a.
3. Wang, X.Y.; Yang, J.Y.; Wang, Y.T.; Zhang, H.C.; Chen, M.L.; Yang, T.; Wang, J.H. M13 phage-based nanoprobe for SERS detection and inactivation of Staphylococcus aureus. *Talanta* **2021**, *221*, 121668, doi:10.1016/j.talanta.2020.121668.
4. Yang, T.; Zhang, X.X.; Yang, J.Y.; Wang, Y.T.; Chen, M.L. Screening arsenic(III)-binding peptide for colorimetric detection of arsenic(III) based on the peptide induced aggregation of gold nanoparticles. *Talanta* **2018**, *177*, 212-216, doi:10.1016/j.talanta.2017.07.005.
